# Internet Applications for Screening and Brief Interventions for Alcohol in Primary Care Settings – Implementation and Sustainability

**DOI:** 10.3389/fpsyt.2014.00151

**Published:** 2014-10-30

**Authors:** Paul Wallace, Preben Bendtsen

**Affiliations:** ^1^University College London, London, UK; ^2^Department of Medical Specialist and Department of Medicine and Health Sciences, Linköping University, Motala, Sweden

**Keywords:** eSBI, online interventions, alcohol, health care setting, Internet, digital

## Abstract

Screening and brief interventions head the list of effective evidence-based interventions for the prevention and treatment of alcohol use disorders in healthcare settings. However, healthcare professionals have been reluctant to engage with this kind of activity both because of the sensitive nature of the subject and because delivery is potentially time-consuming. Digital technologies for behavioral change are becoming increasingly widespread and their low delivery costs make them highly attractive. Internet and mobile technologies have been shown to be effective for the treatment of depression, anxiety, and smoking cessation in healthcare settings, and have the potential to add substantial value to the delivery of brief intervention for alcohol. Online alcohol questionnaires have been shown to elicit reliable responses on alcohol consumption and compared with conventional prevention techniques, digital alcohol interventions delivered in various settings have been found to be as effective in preventing alcohol-related harms. The last decade has seen the emergence of a range of approaches to the implementation in health care settings of referral to Internet-based applications for screening and brief interventions (eSBI) for alcohol. Research in this area is in its infancy, but there is a small body of evidence providing early indications about implementation and sustainability, and a number of studies are currently underway. This paper examines some of the evidence emerging from these and other studies and assesses the implications for the future of eSBI delivery in primary care settings.

## Background

Screening and brief interventions have been demonstrated to be highly cost-effective but despite the strength of the evidence, implementation by healthcare professionals has been disappointingly low (1, 2). The delivery of a standard brief intervention can add up to 15 min to the primary consultation and thus constitutes a significant barrier to implementation. In primary health care settings, commonly <10% of hazardous and harmful drinkers are identified, and <5% of those who could benefit are offered brief interventions (3). Primary health care based interventions for hazardous and harmful alcohol consumption are among the most infrequently delivered interventions when compared with other cost-effective clinical preventive services (4).

Digital technologies for behavioral change are becoming increasingly widespread and their low delivery costs make them highly attractive. Internet and mobile technologies have been shown to be effective for the treatment of depression and anxiety (5–7). Similarly, digital smoking cessation intervention has shown promising results (8–10). Concerning risky alcohol use, there is also a growing evidence base on the effectiveness of electronic screening and brief interventions (eSBI), both in the general population and in university student populations where there is the largest body of evidence (11–16). A review of computer-based interventions (on- and offline) for college drinkers and a meta-analysis of trials on the same subject concluded that computer-delivered interventions reduce the quantity and frequency of drinking in student populations when compared with assessment-only controls, and that computer-based interventions are as effective as other alcohol-related interventions (16, 17).

Several systematic reviews have been undertaken to assess the effectiveness of digital interventions across a wider range of settings (18–20). All concluded that the weight of evidence suggests that users can benefit from online alcohol interventions and that this approach could be particularly useful for groups less likely to access traditional alcohol-related services. However, all also suggested that caution should be exercised in drawing conclusions given the limited number of studies allowing extraction of effect sizes and the heterogeneity of outcome measures and follow-up periods. A recent review by Donoghue et al. identified such a high level of diversity of interventions with regards to length, content, and theoretical basis that it was not practical to perform a meta-analysis of the effectiveness of eSBI (21). Although there is growing evidence for modest effectiveness of a broad range of internet-delivered interventions, a number of issues remain to be resolved (18, 19) with more research still needed into the nature of the active elements of an Internet intervention and the support required to ensure effective use is made of the intervention (19, 20, 22).

So, what can health care staff do in order to facilitate patient referral and uptake of eSBI and to promote the intended use of the intervention? Research on referral of patients by health care professionals to Internet applications for screening and brief interventions for alcohol (eSBI) is still in its infancy, with early studies having been undertaken in Sweden and the UK, and a number of further studies currently underway. These include the ODHIN trial taking place in general practice settings in five European countries, and the EFAR trials, currently in progress in northern Italy and in the planning stages in Spain, Australia, and the UK (23, 24). This paper considers the development of eSBI for delivery in health care settings and the evidence emerging from studies on implementation and uptake, and assesses the implications for the role of eSBI in the future delivery of screening and brief interventions in primary care.

## eSBI Delivered in Primary Care Settings: Case Studies from Sweden and the UK

The majority of studies on eSBI have concentrated on population studies where individuals access these online facilities largely unprompted and of their own volition. These inevitably tend to recruit participants who are self-selected and motivated to engage, presumably because they are actively concerned about their drinking and keen to take action to reduce. There is substantial potential to increase the reach of eSBI beyond these populations by promoting their use in health care settings, particularly primary care where the great majority of health encounters take place. There is already an extensive literature on the effectiveness of face-to-face lifestyle interventions delivered by primary care professionals, which suggests that the interaction between the clinician and patient can be highly effective in bringing about lifestyle change in patients who may not necessarily have made any prior decision to change. There is also a growing literature on guided interventions and facilitated access by health care professionals to online resources for anxiety and depression and risky drinking (5, 25). In a recent meta-review, Riper et al. pointed out that most studies on alcohol eSBI are delivered as stand-alone interventions accessible to the community at large and to a far lesser extent are guided interventions initiated via primary care (25). In the review, no differences in effectiveness were seen between guided and unguided low-intensity eSBI. As has been pointed out by Kohl et al., “one of the most substantial problems in online prevention is the low use of the interventions” (19), and although Brouwer et al. found indications that counselor support may facilitate the uptake of referral to an eSBI (20), more studies are needed on how best to combine face-to-face support with eSBI. We describe below two early initiatives in Sweden and the UK, which were designed to test the feasibility and acceptability of referral to eSBI in primary care settings as an alternative to traditional face-to-face screening and brief interventions.

### Experiences from Sweden

In a series of implementation projects undertaken in Sweden during 2007–2010, the use of stand-alone computers to facilitate automated alcohol screening and personalized feedback to patients was tested in a total of 28 primary care centers (PHCs). For the purposes of the study, a computer-based alcohol screening and brief intervention (eSBI) module was developed. In short, the single-session intervention offered a two pages simple text and graphical based feedback on the persons drinking pattern comparing this with the recommend official sensible drinking limits in Sweden. Suggestions on how to cut down were given based on the person’s motivation to change. A drinking diary for self-monitoring of drinking level was also included in the feedback. While the primary aim of the study was to evaluate the uptake and usefulness of the Internet-based intervention (eBI) module for alcohol, an additional module on physical activity was developed in order to limit stigmatizing patients using the computer (26).

### Piloting the intervention and implementation

In the initial pilot phase, the managers and health coordinators at 9 PHC units were informed in a meeting about the computerized system. Staff in each PHC center was encouraged to decide for themselves, which patients to refer to the computerized test. The 9 PHC units were provided with a stand-alone computer in an integrated IT-kiosk with a touch screen and a printer. The kiosks were placed in the waiting rooms, suitable corridors, or more private rooms at the PHC unit. The staff was given weekly statistics about the number of tests performed and the risk profiles of their patients. All tests were anonymous, but the patients were given two copies of the two pages written feedback in case they wanted to share a copy with the caregiver.

Although the prime objective of the implementation project was for staff members to actively refer patients to the computerized intervention, patients were also free to perform the test without referral. One question in the program asked whether the patients had been referred by a staff member to the computer or had done the test by themselves, and this information was used in the feedback to the staff.

After the first year, a total of 3027 patients had completed the computerized screening module, comprising on average 5.7% of all visitors to the PHC during the period (range 3.6–11.1%). The proportion of patients referred to the intervention by the staff in relation to the total number of tests varied by PHC unit from 11 to 87% of all interventions. A total of 28% of the men and 14% of the women who completed the computer-based screening module had a risky drinking profile. No differences were seen in the proportions of patients with risky drinking between those who had been referred and those who undertook the computer-based screening on their own initiative (26).

### Further implementation and evaluations

A number of evaluations and reports have been published on the pilot study, a subsequent experimental implementation study on 6 PHC units and lastly a larger scale implementation study involving 28 PHC units in the region (26–29). These showed that the patients found the computer-based eSBI module easy to use, irrespective of gender and age, and only 3% of the referred patients expressed negative views about having been referred to the intervention (27). In a follow-up study, 3,169 risky drinkers were invited to participate in a 3 months follow-up after having performed the intervention. Of the 587 patients who agreed to be contacted after 3 months, 347 patients were eventually contacted for follow-up. Of the responders, 84% confirmed that they had read the written feedback that they received on completion of the eSBI module, and 77% stated that they remembered the content. Eighty-two percent agreed that the feedback was relevant, 45% had discussed the feedback with a friend or relative, and 26% had talked about it with someone at the PHC unit. Nearly all (92%) found the information easy to understand (27).

In a sub study on six PHC units, interviews were performed with managers and health care professionals. The managers were unanimously positive about the computerized intervention and saw openness among their staff for this innovation. However, they also indicated more negative attitudes among certain groups of staff, especially the GPs. Interviews with GPs confirmed that there was less enthusiasm in this group, and some pointed out that they had enough to do without the tool. Commonly, the GPs stated that they did not need a new tool as they integrated lifestyle advice into their consultations. This stood in contrast to much more positive attitudes from nurses to the computerized intervention (28).

A follow-up study undertaken 2 years subsequent to the introduction of the computerized intervention into the six PHC units showed that levels of maintenance/sustainability were low. However, most staff agreed that computerized or Internet-based interventions could facilitate healthy lifestyle promotion and that using computers is an important tool for increasing healthy lifestyle promotion (29).

#### In summary

The Swedish experience tells us that the stand-alone computerized intervention may be implemented within the PHC settings in the short term, and succeeded in reaching large numbers of patients. Many patients and some staff expressed generally positive attitudes about the eSBI module. However, there was less enthusiasm among the GPs and importantly usage decreased over time even though many of the staff considered the computerized tool as an important part of the healthy life style promotion. It therefore seems unlikely that this model could be sustainable in routine practice not least due to the low levels of interest among staff in promoting a healthy life style among patient with no obvious risk factors.

### Experiences from the UK

An implementation study was designed to test the feasibility of offering an online self-help alcohol reduction program [Down Your Drink (DYD)] and support from a trained alcohol worker to general practice patients found to have hazardous or harmful drinking (30). The project was carried out in Kingston Primary Care Trust, which is situated on the outskirts of London, UK. For the duration of the study, an alcohol project co-ordinator (APC) was employed to work with the risky drinkers identified by GPs and nurses in the participating practices, and to help them use DYD in order to reduce their drinking. Once the GPs and practice nurses identified a patient with hazardous or harmful consumption, they referred the patient to the APC, who then contacted the patient and arranged an appointment. The APC explained the nature of DYD to the patient, provided an introduction to the various sections of the intervention, and gave the patient personalized login details. The patient was then invited to log in to the intervention from their own computer. On average, this appointment lasted just over 40 min. Patients were able to call the APC if they experienced any difficulties using the web site, and the APC arranged three follow-up phone calls at fortnightly intervals in order to ensure that patients were succeeding in using the website. They did not engage in counseling.

A total of 18 of 28 practices expressed an interest in referring patients to the web-based intervention, but after 12 months, only 31 patients had been referred to the service of whom 19 attended the appointment with the APC. Only 6 of these 19 patients seen by the APC subsequently logged on to the web-based intervention. However, those who chose to use the web site did this to a high degree, with a mean of eight log-ins per patient and a mean of 13 pages visited per session. Interviews were subsequently performed with patients and the health care staff in the participating practices, and these suggested that both staff and patients found the service highly acceptable. They also reported that the service worked smoothly and that it was convenient being able to access the intervention in their own time.

#### In summary

The English experience of referring patients to a web-based self-help alcohol intervention suggested that where practices are willing to innovate, implementation is feasible and it is generally acceptable to both staff and patients. There were, however, few referrals, probably in part as a consequence of low levels of screening for hazardous and harmful drinking, which is typical of primary care. The cost of running the service was high due to the amount of time that the APC spent with each patient, and it seems unlikely that this model could be sustainable in routine practice.

## Facilitated Access to eSBI in Primary Care Settings: the ODHIN Study and EFAR Studies

These early studies in Sweden and the UK suggested that while eSBI might have potential to supplement conventional face-to-face approaches to SBI in primary care settings, more attention clearly needed to be given to make the technology sustainable. Research was also required to determine whether eBI was as effective as face-to-face intervention in these settings. The following section examines two sets of studies designed to determine how simple referral of patients by GPs and nurses to an eSBI website (“facilitated access”) might affect screening and brief intervention activity in primary care settings (the ODHIN study), and whether this approach could be as effective as face-to-face intervention (the EFAR studies).

### The ODHIN study

The ODHIN Study (Optimizing Delivery of Health Care Interventions) is a Europe wide project designed to help to optimize the delivery of health care interventions by understanding how better to translate the results of clinical research into every day practice (23). ODHIN uses hazardous and harmful alcohol consumption in primary health care as a case study to investigate the implementation of identification and brief intervention program.

The ODHIN study is being undertaken in Spain, England, the Netherlands, Poland, and Sweden. In each country, 24 PHCs are participating in a cluster randomized study with eight arms. The aim is to study the effectiveness of training and support, financial reimbursement, and referral to an eBI targeted singly or in combination. Each country referred to existing Internet-based interventions already in use in the particular country. This meant that in Sweden, the patients were referred to a single-session intervention similar to the one described previously in this paper. In the UK, the patients were referred to a modified version of “DYD.” In the Netherlands, the “Minderdrinken.nl” web site was used. In Spain, the patients were referred to an existing web site run by the Ministry of Health. In Poland, a new web site developed by the WHO was used.

For all arms of the trial, staff in participating practices are encouraged to use a short screening questionnaire administered face-to-face to screen their patients for hazardous and harmful drinking and to subsequently deliver brief interventions to those who screen positive. In the eBI condition, the health care professionals have the option to actively refer patients who screen positive to the online resource as an alternative to offering a face-to-face intervention. In this case, the health care professional is asked to spend 2–3 min providing the patient with facilitated access to the alcohol reduction website, including a short conversation and the offer of a leaflet offering information about the website, encouragement to log-on, and a personalized login code. The doctors and nurses participating in the study participating in the study are instructed to follow a script when offering facilitated access, including a negotiation on when the patient thinks that he/she would have time to log-on. The patients are handed a short leaflet informing them why they have been referred, and on this leaflet there are also given a personal login code, which can subsequently be used to track log in activity.

The study is still in progress and the definitive results are expected to be available in 2015. However, the preliminary data indicate that facilitated access activity was highly variable across the participating practices randomized to this arm of the trial. Furthermore, it appears that while for some staff a high proportion of the patients they refer to eBI complete the log-in process, for others only a few do so. Such variability is almost certainly due at least in part to the nature of the facilitated access provided by each individual health care professional to their patients.

#### In summary

This model appears to hold significant promise as it has the advantage that it does not require any additional staff. However, effective implementation will probably depend critically on ensuring consistency among staff in the delivery of facilitated access to the web-based intervention. Furthermore, studies need to be performed in order to explore whether facilitated access at the index consultation is sufficient to ensure that patients to make adequate use of the service, or whether active follow-up is needed. Also, it is unclear to what extent staff want or need a feedback on the effects of the web-based intervention in order to sustain motivated to the use of the referral system.

### The EFAR studies

The EFAR studies form part of a multi-country initiative involving a series of randomized controlled non-inferiority trials of primary care based facilitated access to an alcohol reduction website, which are at various stages of development in Italy, Spain, Australia, and the UK. The EFAR-FVG trial is being undertaken in general practices in the Friuli Venezia Giulia Region of Italy and is the first in the series (24). EFAR-FVG compares delivery by GPs of facilitated access to a dedicated website for risky drinkers with standard face-to-face brief intervention. The trial website is an Italian language online facility, which includes modules for all the key trial components including screening, consent, assessment, randomization, and follow-up. It also incorporates the alcohol reduction website for the patients in the experimental group. The site has been adapted from the website developed for the DYD-RCT trial. Details of the DYD website and the psychological theory, which has underpinned its development, have been reported elsewhere (31). The EFAR-FVG trial website additionally incorporates a menu-driven facility to enable the GPs to personalize the automated patient messages by adding a photograph of themselves and/or an audio/video recorded message.

All patients aged 18 years or over who attend the participating practices are offered facilitated access to the eSBI facility by their GP or another staff member. This consists of a 2–3 min discussion followed by the offer of a trial brochure providing a unique access number enabling the patient to log on to the trial website from their own computer. Once online, patients are asked to complete the three-question short Alcohol Use Disorders Identification Test (AUDIT-C) and to provide agreement for the results of the test to be sent to their practice. For the purposes of the trial, cut points of four for women and five for men have been used. Those scoring at or above the cut points receive personalized feedback advising that their stated drinking patterns indicate that they are at risk from their drinking and inviting them to take part in the study. They are then invited to complete the online EFAR-FVG trial consent module before being invited to complete the online baseline assessment, which includes the full AUDIT, and two brief questionnaires on demographics and quality of life. Completion of the questionnaires leads to automated online randomization to either online (experimental) or face-to-face (reference) intervention.

Those in the experimental group are greeted by a personalized online message from their GP with tailored feedback about their responses to the questionnaires and encouragement to spend some time online to consider their alcohol consumption and ways to reduce. Patients receive an email 1 week later encouraging them to log on again. They are also asked online to review their alcohol consumption and are invited to discuss their website experience when they next see their GP. Patients allocated to the standard intervention group are invited to check a box online, which automatically generates an email to their practice requesting a GP appointment for a face-to-face brief intervention within the next 7–10 days.

The findings of the pilot study indicated that this approach was acceptable both to the participating GPs and the patients receiving facilitated access (unpublished data). The numbers of brochures distributed by each GP ranged between 22 and 280, and on average 42% of the patients receiving a brochure subsequently logged on to the website. Of these, 93% completed the screening questionnaire, and of the 20% who screened positive, 84% went on to randomization. The main trial, which commenced in January 2014, has recruited in excess of 500 patients and is due to report in 2015.

#### In summary

The facilitated access approach adopted in the EFAR-FVG study appears to have been generally effective in achieving relatively high rates of online screening in the participating practices, though the rates varied considerably between GPs. The results of the relative effectiveness of facilitated access to eSBI compared with face-to-face intervention are yet to be published. The other EFAR trials will examine additional approaches to practice based eSBI, such as the use of a digital tablet in the practice and whether the GP personalization facility in the alcohol reduction website adds to the effectiveness of the online intervention. The results of these trials will give an indication about whether GP facilitated access to an alcohol reduction website is as effective as face-to-face, and while the issue of sustainability will not be directly addressed, useful indications about this might well emerge from add-on studies conducted after the conclusion of the trials.

## Discussion

The last decade has seen the emergence of a range of approaches to the delivery of eSBI as an integrated part of primary health care settings as outlined in this paper, most of which have been undertaken in European settings. Experimentation of this approach is also being undertaken in other settings (32, 33) and we are aware of at least one study of electronic screening currently taking place in accident and emergency departments in the UK as part of the SIPS Junior study (http://www.sipsjunior.net/).

To date, the literature on evaluation of implementation of referral to eSBI is limited, but in our view there are grounds for cautious optimism about the potential for implementation and sustainability in primary health care settings. For example, there is reasonable evidence about the potential for internet applications to increase alcohol screening rates. This includes the findings from the Swedish study where more than 3000 primary health care patients (5.7% of all visitors to the PHC) used the computerized screening facility in the space of year (26), and from the DYD trial in the general population where high screening rates were achieved with more than 10,000 screen positive subjects identified over the course of the trial. (31) Additionally, in the EFAR trial more than 40% of all the patients who received facilitated access from their GP went on to complete the online alcohol screening module (unpublished data). There thus appears to be real potential for internet applications substantially to increase alcohol screening rates. Given the consistently low screening rates, which have been achieved by conventional approaches, it would appear to be reasonable to advocate the more widespread implementation of online screening in primary health care settings. This might be achieved either through the direct provision in the clinic of access to a screening module using a computer or a tablet, or by the GP or other health professional facilitating their patients’ access to a suitable internet application through provision of website details and/or log-on codes. There are as yet unanswered questions about how the results of each patient’s alcohol screening test should best be fed back to the relevant health care professional, though in any case, patients will need to be reassured that their data will be treated in strict confidence and notification should be conditional on provision of consent. Ideally automated electronic transmission should be used to transfer the screening results direct to the patient’s care record, but the wide variety of primary health care systems providers operating in most countries poses significant technical challenges to this approach, and thus more simple procedures using printout maybe more practical.

While we believe that the evidence on internet applications for screening in primary healthcare settings is encouraging, we feel that the case for using these applications for brief intervention (eBI) is currently less clear cut. Overall, there appears to be evidence that internet applications may be as effective as face-to-face intervention for risky drinkers, especially for student populations. (16, 17) However, a number of authors of systematic reviews have cautioned over-interpretation of the findings from the literature, given the predominance of small-scale studies of variable quality. The evidence for the use of eBI in health care settings is certainly not yet convincing, but as highlighted in this paper, there are some indications of promise for this approach, and a number of important studies are now underway, which should provide better evidence. The UK study of referral to eBI following face-to-face screening was too small to enable firm conclusions to be drawn (30). The results from the ODHIN study, which are still to be published, should provide more robust evidence on the impact of providing health care professionals with the option to use facilitated access to an internet application for patients who have screened positive on direct testing with the AUDIT-C. However, the study will not provide any evidence of effectiveness of eBI relative to face-to-face intervention, and in the absence of other studies in this area, we will need to await the results of the EFAR trials before this question can be answered.

Taking all of these factors into account, we believe that there is a good case for advocating the more widespread use of or referral to internet applications for alcohol screening in primary healthcare settings. We think that the same is likely to be true for internet applications for brief intervention, though robust evidence on this is still awaited. Whatever the case, it is clear that sustainability will depend on appropriate configuration to meet the needs and wishes of both patients and healthcare professionals, and the degree to which these interventions become embedded in everyday practice will depend critically on the way in which their implementation is configured. Although many patients appear to respond positively to advice from their health care professional to undertake eSBI, the professionals themselves demonstrate much more variable levels of engagement. Effective mechanisms will therefore be needed to enable referral to internet applications for screening and brief intervention to become embedded in professional practice. The challenges involved are complex and relate not only to considerations of whether patients and healthcare professionals are prepared to place their trust in internet applications but also to the general attitude of healthcare professionals toward working with alcohol and other life style areas.

## Conclusion

If an appropriate balance can be identified between the use of referral to the internet and the personal engagement of the healthcare professional, it is likely that the use of internet applications for alcohol screening and brief advice will prove increasingly successful. Indeed, this approach may well find a growing range of applications beyond alcohol not only for other lifestyle behaviors but also for the management of long term conditions such as asthma, diabetes, and arthritis. There is thus real potential to develop integrated virtual healthcare environments designed to complement face-to-face delivery of health care and capable of providing patients with access to internet applications for a growing range of their health care needs. As with eSBI, their success will depend critically on our ability to identify the key elements, which contribute not only to effectiveness but also to sustainability.

## Author Contributions

Both authors contributed to the design and content of the manuscript as well as the first draft of the manuscript and all subsequent revisions. Both authors have approved the final version of the manuscript.

## Conflict of Interest Statement

Preben Bendtsen partly owns a private company that distributes alcohol and other life style online interventions. Paul Wallace has intellectual property rights for www.downyourdrink.org.uk, is PI in the EFAR and ODHIN studies and provides private consultancy on the topic of screening and brief interventions.
